# National charitable fundraising for the NHS, 1948–2023

**DOI:** 10.1111/spol.13049

**Published:** 2024-07-01

**Authors:** Ellen Stewart, Rosemary Cresswell, Christian Möller

**Affiliations:** ^1^ University of Glasgow Glasgow UK; ^2^ University of Strathclyde Glasgow UK

**Keywords:** health care systems, health care, economics of, health policy

## Abstract

Whether charitable fundraising might play a part in funding Britain's ostensibly tax‐funded NHS has been a longstanding dilemma, which until recently has received only occasional scholarly attention. In 1946, Aneurin Bevan argued that one of the main goals of the reformed health care system was to liberate health care from the ‘caprice of private charity’. Seven decades later, NHS Charities Together's Urgent Covid‐19 Appeal became a powerful societal rallying cry in the health emergency of the pandemic and raised £150 million in the process. This paper draws together findings from new archival research, a witness seminar with key actors in the NHS charity sector, and qualitative research based on interviews with NHS charity staff and trustees (*N* = 13), all conducted between 2021 and 2023. We investigate the way in which *national* appeals have been proposed, debated and implemented at different times in the NHS's history. We trace the recurrence of conflicting ideas about the acceptability of national fundraising for the NHS, about whether public loyalties are to their local services or the national ‘brand’ and about the introduction of national appeals into a complex ecology of local NHS charities. The history of charitable fundraising for the NHS is, we argue, neither a simple story of spontaneous public generosity, nor often of formal policy reform, but is an artefact of more complex dynamics between a changing cast of local and national actors over the last 75 years.

## 1 INTRODUCTION

Financing has been one of the UK NHS's over‐arching challenges, and a recurring focus for reviews and Commissions over its decades (Powell, 2018). The role of charitable donations in funding the predominantly tax‐funded NHS, is a similarly longstanding policy dilemma that has received far less scholarly attention. In 1946, Bevan argued that one of the main goals of creating the NHS was to liberate healthcare from the ‘caprice of private charity’ and that it was ‘repugnant to a civilised community for hospitals to have to rely upon private charity’ (HC Deb, 30 April 1946, cols. 46‐47). Seven decades later, NHS Charities Together's Urgent Covid‐19 Appeal became a powerful societal rallying cry in the health emergency of the pandemic, and raised £150 million in the process (Stewart et al., 2022). This paper shares new archival and contemporary qualitative research to explore this apparent transformation, with particular focus on calls for and attempts at *national* NHS campaigns, and their interaction with longer standing traditions of local hospital fundraising.

While an extensive landscape of health charities make contributions to healthcare in the UK, our focus in this paper is more specifically on how charitable fundraising has developed within the NHS, and in the cohort of NHS charities which originated within, and are still primarily focused on donations to, a single NHS organisation. As well as providing what can be considered ‘nice extras’, charitable funding has always contributed to ‘core’ statutory provision in the NHS, albeit in a highly uneven fashion across the country. Examples include contributing to medical equipment, buildings, and things considered ‘comforts’ (such as comfortable chairs for the bedside) (Abnett et al., 2023; Lattimer, 1996). Especially since a key policy change in the 1980s (the Health Services Act 1980, which amended the 1977 Act to enable fundraising), the bulk of charitable activity in the NHS has been local. NHS hospitals brought very unequal inherited wealth from their pre‐NHS existence into the new system (Mohan & Gorsky, 2001), as large voluntary teaching hospitals were permitted to retain their pre‐NHS endowments, with some policy differences in Scotland and Northern Ireland (Harris & Cresswell, 2024). The 1980 liberalisation of rules around NHS fundraising contributed to an uneven expansion of local efforts across the country, and an emerging evidence base suggests that, especially in England, local NHS fundraising risks entrenching spatial inequalities between different areas (Abnett et al., 2023; Bowles et al., 2023).

Acknowledging this growing scholarship, this paper investigates instead the way in which *national* appeals have been proposed, debated and implemented at different times in the NHS's 75‐year history. We will demonstrate that in the early years of the NHS there was policy tolerance for regional or national^1^ appeals, but that hospital staff were dissuaded from playing an active role in local appeals. Over the decades, acceptance of fundraising to supplement Exchequer funding for the NHS grew, and the 1980 legislative changes enabled an expansion of local fundraising by (and thus competition between) individual NHS organisations, especially in London. Despite proposals around a National Hospital Trust NHS lottery from 1988, it was not until planning around the celebration of the 70th anniversary of the NHS in 2018 that the first official NHS‐branded fundraising campaign took place, and this was instigated by NHS England, rather than local NHS charities. At the 75th anniversary, galvanised by COVID‐19 fundraising, the NHS charity sector is transformed into a significant actor, rather than a more disparate collection of distinctive organisations (Möller & Abnett, 2023).

Social policy scholarship on charity within the mixed economy of welfare emphasises that charitable contributions are not simply a spontaneous response to the inadequacy of state provision, but instead are entangled with dominant patterns of financing and provision (Parsell et al., 2021). Reich describes this as understanding philanthropy as ‘not an invention of the state… but an artifact of the state’ (Reich, 2020). As such, national fundraising (and stymied proposals for it), shed new light on what Exworthy, Mannion, and Powell (2023) identify as one of the key ‘storylines’ of the NHS; efforts to centralise, or decentralise power within the system. We argue that, while Governments have neither invented, nor paid a great deal of attention to, the role of charitable fundraising in the NHS, the ebbs and flows of efforts at national fundraising projects nonetheless reveal insights about health policy in the UK. Decisions about national or local NHS fundraising have implications both for the balance of local discretion and central control and, relatedly, for whether public support is mobilised towards local hospitals or a more national vision of the NHS.

## 2 CHARITY IN THE MIXED ECONOMY OF HEALTHCARE: FOR BETTER OR WORSE

The role of charity within mixed economies of healthcare has been subject to both critiques and defence; and arguments from studies of charity in the broader welfare state also have relevance. Critiques of charity in advanced welfare states argue that by mitigating the immediate consequences of state retreat, charity distracts or ‘soothes’ from poverty's more structural determinants (Parsell et al., 2021). The growth of food banks has become a totemic, and increasingly controversial, example of this (Möller, 2021; Power, 2022). Focusing more narrowly on charity in healthcare, a key objection is that by allowing the wealthy to enhance their local services, discretionary financial donations may exacerbate the sort of geographical inequalities that universal public services seek to redress (Bowles et al., 2023). The ‘caprice’ that Bevan cited—the uncertain, unpredictable and short‐term nature of charitable funding—might not lend itself to pressing and long‐term population level challenges (Lattimer, 1996). Pursuing charitable funding might influence organisational behaviour in undesirable ways: in the English NHS, Hodgson et al. (2022) argue that charitable activities can act as a palatable gateway to the further commercialisation of NHS organisations via both corporate donations and a more diffuse expansion of branding and marketing in service of NHS charity.

There are also advantageous aspects to charitable fundraising in a welfare state. These have been less discussed in scholarly discussion on the NHS, where charitable funds have been seen as a sub‐set of private finance, rather than a distinctive phenomenon (Ruane, 1997). Fundraising can be understood as a route to community participation in healthcare, helping develop more person and community‐centred provision in a counterweight to medical or managerial power (Stewart, 2021), especially in an era where ‘community‐powered healthcare’ is gaining traction (Lent et al., 2022). Many fundraising activities (such as sponsored sporting events, fundraising fairs and events) are themselves now seen as broadly beneficial for health and wellbeing, by building community and reducing social isolation, notwithstanding studies which suggest NHS spending on treating accidents from sponsored parachute jumps outweighed the sums raised (Jessop, 1985; Lee et al., 1999). Despite the fear that charity distracts from structural causes of scarcity, community connections forged through fundraising might also generate new spaces of political critique. In the field of food poverty, research has identified that the mutual aid character of some charitable efforts (Cloke et al., 2017) also creates potential for more critical encounters between community members (Cloke et al., 2017; Power & Small, 2022) which might strengthen societal solidarity and encourage resistance to the dilution of the welfare state. While our goal in this paper is not narrowly evaluative, these contrasting rationales and objections to the role of charitable fundraising within the mixed economies of healthcare and welfare, are highly pertinent for an understanding of how proposals to expand national NHS fundraising have fared over the last 75 years.

## 3 METHODS

We draw on a combination of historical and social science methods (see Table 1), conducted as part of a major collaborative research project on the past and present of charitable initiative within the NHS. Historical research, led by Cresswell, has utilised archival and published sources, particularly from the UK's National Archives in Kew, London, the London Metropolitan Archives, and from Northern Ireland including those held at the Healthcare Library of Northern Ireland at Queen's University, Belfast.

Contemporary material comes from two datasets. Möller conducted semi‐structured interviews with 9 senior staff and trustees of NHS Charities Together between 2021 and 2023. Interviews covered participants' careers, current roles and their hopes for the future of NHS fundraising. In addition, an online focus group and interview with staff from four NHS charities were conducted in 2022 by Stewart and Möller to discuss their involvement and experience with the ‘Big Tea’ national fundraiser. Interviews and the focus group were audio recorded, transcribed and transcripts shared with participants for approval or amendments. Transcripts were imported into NVivo and thematically coded by Möller, in discussion with Stewart.

Finally, we draw upon the transcript of a Witness Seminar with 9 NHS and League of Friends fundraisers who had worked in the sector between 1980 and the present day, held in London in 2023. Witness Seminars are group oral histories and question and answer sessions with an invited audience. The conversation was audio recorded, transcribed, and the transcript approved by all speakers. The terms of our ethics approval for interviews (from University of Strathclyde School of Social Policy & Social Work Ethics Committee) requires us to anonymise all quotes from interviews, and by contrast the terms of the ethics approval for the witness seminar (from London School of Hygiene & Tropical Medicine) requires identification of the speaker when quoting. We see this difference in approach as one of the compromises of multidisciplinary research, and hope the paper also demonstrates some of the advantages thereof.

**TABLE 1:** Data sources for this paper.

Data source
Archival records of Government, charity and NHS organisations
Semi‐structured interviews and focus group with 13 current and recent NHS Charities staff and trustees, conducted between 2021 and 2023
Transcript of Witness Seminar with NHS fundraisers from 1980s to 2023, held in 2023

## 4 FINDINGS

### 4.1 The first decades

The transition from voluntary hospitals to state‐funded NHS hospitals involved some complex negotiations around fundraising activities (Harris & Cresswell, 2024; Piggott, 2022). Generally, local fundraising by hospital staff and committee members themselves was dissuaded. Voluntary groups known as Leagues of Friends retained the ability to fundraise for hospitals as long as they did so outside the hospital premises (Ministry of Health, 1948), and became an important resource for volunteering time and funding (Millward, 2023). The church‐led activity, Hospital Sunday, continued to raise funds but directed them to hospitals via Leagues of Friends following 1948. A national version of Hospital Sunday was attempted, with only partial success, around St Luke's Tide in October (Piggott, 2022). There were deliberate efforts from Government to mark a break from the voluntaristic origins of hospitals. Hospital staff or committee members could not overtly be involved in fundraising; they should not appear in uniform, nor in public (Ministry of Health, 1948). In 1952, there was some discussion about the relaxation of these rules to informally permit Christmas collection boxes in England and Wales (Ministry of Health, 1952), and by the 1960s, hospital staff in Northern Ireland were actively involved in fundraising for a hospital minibus (Northern Ireland Hospital Authority, 1964).

While the rules on local fundraising by hospitals' staff and committee members were initially restrictive, fundraising *was* allowed for charities which operated at regional and national level, as long as funds were not collected for a particular hospital (Ministry of Health, 1949). The most prominent example—King Edward's Hospital Fund for London—could still fundraise but opted not to, having enough resources because of large donations (Prochaska, 1997). Yet the King's Fund continued to award grants to NHS hospitals (Prochaska, 1997). Another fundraising campaign which donated money to NHS hospitals was Alexandra Rose Day which had, since 1912, marked the anniversary of Queen Alexandra's 1862 arrival in the UK. Real, and then subsequently artificial, roses were sold to raise funds for Londoners and hospitals. It became an annual national event although funds were raised and kept in local areas, with a contribution to the national office from each local area (Alexandra Rose Charities, 1949). Doyle's (2014) research on Yorkshire reveals that street collections such as Alexandra Rose Day had already declined in popularity by the early 1930s. However the new NHS had a further impact, and while Alexandra Rose Day continued to raise funds for hospitals after the launch of the NHS, sums raised dropped significantly after 1948 (Alexandra Rose Charities, 1949). The Alexandra Rose Day executive committee decided to focus instead on purposes such as care for the elderly that were not covered by the NHS, although fundraising for NHS hospitals' Christmas Funds was mentioned in 1949, and grants of a maximum of £50 to hospitals' amenities funds were noted in 1951 (Alexandra Rose Charities, 1949, 1951). Nowadays, the organisation pursues health causes by providing ‘Rose Vouchers for Fruit and Veg’ for families on low incomes. These examples demonstrate first, negotiations within and between existing regional and national appeals for hospitals following the creation of the NHS, and second that appeals at this scale *were* permitted to continue contributing to NHS causes, even as local hospital staff were dissuaded from doing so. The objection to hospital fundraising was, this suggests, not a principled objection to charitable contributions in general, but a concern with local NHS hospitals *appearing* to be in financial need.

Continued anxiety about funding the NHS prompted calls for a very different mode of charitable fundraising—national sweepstakes and lotteries—from the 1960s onwards. While this might seem incongruous, given public health concerns about gambling harms, it was a recurrent proposal. William Hamilton MP (Labour Party), proposed a national lottery for the NHS in the House of Commons in 1965 suggesting ‘unorthodox policies’ were needed in order to be able to fund hospital building and modernisation (HC Deb, 14 December 1965, cols. 1060‐1061). In 1966, the *Daily Mail* (McLeave, 1966) reported ‘Doctors want hospital sweep’, claiming that the British Medical Association suggested the idea of a hospital sweepstake. The National Union of Townswomen's Guilds also passed a resolution in favour of a national hospital sweepstake in the same year (Williams, 1966). The idea of a lottery was mooted again in a Home Office Report published in December 1973 (Groser, 1973), and the Lotteries and Amusements Act 1976 permitted local organisations including Leagues of Friends, but not health authorities, to use lotteries for fundraising. In 1988 Prime Minster Margaret Thatcher was initially positive about proposals for the National Health Lottery (HC Deb, 21 April 1988, col. 984; Brown, 1988): a group of local lotteries run by Loto Ltd with funds raised to be distributed by the National Hospital Trust, headed by a former president of the Royal College of Physicians (Home Office, 1988). However, this was soon declared illegal as only local lotteries were permitted by the 1976 Act (HC Deb, 7 June 1988, Written Answers), and in 1988 Statutory Instrument 1988 No. 2161 made this clear. An unofficial ‘NHS Loto’ was launched in 1995, without Department of Health endorsement; when queried in Parliament, it was stated that ‘The use of the letters NHS is not the subject of any express provisions in legislations’ (HC Deb, 10 July 1995, Written Answers). This is in sharp contrast to efforts made in the 1990s, still relevant today, to not only consolidate but protect NHS branding from unofficial uses (Thomson, 2021).

During the 1980s, some hospitals also launched fundraising campaigns at an unprecedented scale, which cannot reasonably be considered ‘local’ appeals. As Government policy had turned away from top‐down hospital planning towards a more decentralised landscape of hospitals (Klein, 1981), and fundraising regulations for local hospitals were relaxed, several large hospitals launched major appeals. The key example was Great Ormond Street Children's Hospital's Wishing Well appeal, which operated at a scale which drew money from across the whole country. As Marion Allford, who led the Wishing Well Appeal, described, this could at times cause conflict with other hospitals:

We set up a whole lot of regional [fundraising] groups and of course they might have been on the doorstep of another children's hospital and they didn't like it. So we did have problems. We had to negotiate arrangements, because they felt we were on their patch, but being a national hospital, how could we do it otherwise? (Gorsky & Arnold‐Forster, 2024)

The ambition and success of the Wishing Well Appeal went on to inspire other large‐scale capital appeals from other hospitals (Gorsky & Arnold‐Forster, 2024). Several decades later, this baseline of professional and highly successful major appeals from hospitals within the NHS set the stage for the first NHS‐branded national appeals in the 2010s.

### 4.2 National campaigns and the NHS 70th birthday

Despite calls for lotteries to address the NHS's funding issues, the advent of official *national* fundraising for the NHS under ‘NHS’ branding, did not come to fruition until planning for the 70th anniversary of the creation of the NHS, celebrated in 2018. Bivins and Thomson (2023), tracing the history of whether and how anniversaries of ‘the appointed day’ have been marked, identify 2018 as something of a turning point in the tenor of such events:

The 70th anniversary of 2018 took celebration to a new level. This had not just the powerful institutional support of the BBC and broader media behind it, but also the backing of politicians and NHS leadership (Bivins & Thomson, 2023).

Interviewees told us that it was NHS England who approached the Association of NHS Charities and suggested a national fundraising campaign in the run up to the 70th anniversary. NHS England was created as an arms' length body in the 2012 Lansley reforms, and quickly became ‘the Mother of all Non Departmental Bodies’ (Rutter, 2014) by virtue of its scale and power. By 2017 it had acquired a reputation for vigorously pursuing its strategic objectives with surprisingly little reference to Government or existing legislation (Hammond et al., 2019). The idea of a national fundraising campaign was one of a range of official celebration activities being led by NHS England. Taken together, these activities can be seen as a strategic mobilisation of public support for the NHS, shortly before a major financial settlement in which Prime Minister Theresa May pledged an increase of over £20 billion in NHS England's budget between 2018 and 2023 (National Audit Office, 2019).

The idea of a national campaign was a major departure for the NHS charity sector, and the umbrella organisation Association of NHS Charities did not take NHS England's suggestion forward. One of our interviewees described the Association's response as: ‘don't want to know, we don't do national things, we don't do fundraising, we don't get involved in things like this, we're a membership organisation, it's too political’ (interview 2023004). Leaders of a number of the largest NHS charities had for some years been informally meeting as ‘the Maddox group’, and had under this name commissioned work including a report from New Philanthropy Capital promoting more professionalised approaches to evaluating the work of NHS charities (New Philanthropy Capital, 2016). It was this group of leaders from some of the most professionalised of the NHS charities who took up the mantle of a national campaign for the 70th birthday. Crucially, the initial Big Tea *wasn't* run by the Association of NHS Charities, but operated independently by key players from the Maddox Group, who coined the term ‘NHS Charities Together’ in order to formally request permission from the Secretary of State to use the NHS trademark.

Despite not being an official initiative of the Association of NHS Charities, the first Big Tea in 2018 (the Big 7Tea, as it was known) was designed to be open to any NHS charities who wanted to be involved. The Maddox Group employed a professional marketing agency to build a website and design some resources for local charities to use, and about 100 took part in the first year. The website created a single front door for the Big Tea, through which:

you could go onto that, see if there was stuff in your area and if there wasn't or if you wanted you could donate to your local hospital or you could donate to NHS charities collectively, which is kind of in a funny sort of way the start of NHS Charities Together fundraising. (interview 2023004)

The success of this initial campaign was measured not in money raised, but in positive visibility for both member charities and, crucially, the sector as a whole.

Now, it didn't raise very much money and it was never really, to be fair, seen as a massive fundraiser. It was seen as a way to mark the birthday, to get some NHS charities a bit of visibility and to show that we could work together. (interview 2023004)

This cooperation was far from unanimous, but concerns were perceived to be motivated by fear of competition from national fundraising—that mobilising the NHS brand would be too successful. Key people involved in creating the first Big Tea, all of whom led already successful NHS charities, explicitly described it as an act of generosity from the larger charities within the sector towards smaller organisations who might not be actively fundraising:

while we might not have got as much out of it as others, it was one of the things you did as a large NHS charity…traditionally all the large NHS charities were members of the association and we felt that was part of our appeal and part of our support we had to give the whole sector. (interview 2021002)

The Big Tea thus spawned a national fundraiser but also laid the groundwork for a transformation of the Association of NHS Charities in the late 2010s into a much more active force within the NHS.

### 4.3 National campaigns in the COVID‐19 pandemic

From the outside NHS Charities Together's Urgent COVID‐19 appeal—which eventually raised over £150 million for NHS charities and sharply increased public awareness of the sector—seemed to spring from nowhere. However, this campaign was rooted in years of work which began following the Big Tea in 2018, when the membership association changed leadership and launched an ambitious new strategy which centred both on increasing national fundraising, and a parallel desire to broaden and strengthen the membership of the national organisation. A national campaigns sub‐committee of NHS Charities Together, including representatives from member charities and NHS England, was due to meet on 17th March in a regular planning meeting for the Big Tea campaign. In a pre‐meeting senior NHSCT staff agreed that the Big Tea model was inappropriate under COVID‐19 social distancing rules.

We agreed—no, we're not doing that. [Trustee] said ‘do you think we should do a national appeal to support the NHS through Covid’ and I went ‘yes, we should. And the money needs to come directly into NHS Charities Together, because this is going to be an urgent appeal and it needs to be simple’. (NHSCT Chief Executive, quoted in Gorsky & Arnold‐Forster, 2024)

This decision was to have ramifications for the scale and structure of national fundraising for the NHS through the pandemic. It was, as the Chief Executive clearly states, one based on the experience of the difficulties of communicating the national/local dimension of NHS fundraising in the Big Tea: ‘trying to tell people that they could give to 1 of 130 charities… is very complex and I'm not sure how well that works’ (NHSCT Chief Executive, quoted in Gorsky & Arnold‐Forster, 2024).

The argument that the simpler, and more national, character of the urgent appeal galvanised support and donations, seems unarguable. In practice, other research has suggested that the simplicity could yield vagueness, and that, especially in early examples of public fundraising via crowdfunding platforms, it was often not clear what people were fundraising for, beyond a generalised sense of gratitude (Stewart et al., 2022). Nonetheless this enabled the appeal to operate at a far greater reach (in terms of numbers of participants) and scale (in terms of money raised) than anticipated: ‘Honestly at the time we thought we'd maybe raise probably tens of thousands of pounds’ (NHSCT Chief Executive, quoted in Gorsky & Arnold‐Forster, 2024). While the scale of community fundraising (and especially the efforts of veteran and fundraiser ‘Captain Tom’) was a significant part of the visibility of the appeal, its financial clout was significantly increased by the corporate engagements that a national appeal made possible:

There was a recognition that having NHS Charities Together as the conduit for the national outpouring of love there was at that time for the NHS was very useful, and there is absolutely no way that Captain Tom's money or any of those other big fundraising initiatives or the big gifts from corporates and high‐net‐worths would have gone to one charity. (NHSCT Chief Executive, quoted in Gorsky & Arnold‐Forster, 2024)

The success of the unified national campaign had two significant consequences, which, intertwined, yielded a shift in the sector. A first is national/local dynamics between NHSCT and the membership of local NHS charities. The success of the appeal, and the money which NHSCT then disbursed to members, was transformative for the membership. There remains some worry from some members that the strengthened NHSCT might supplant, and not complement, local fundraising efforts. However, many new members also joined: membership increased sharply from about 140 members in 2018 to over 230 in 2023. NHSCT disbursed the funds from the Urgent Appeal in three waves of funding: first calculated per head of staff within the NHS trust, and then on application from charities, but with funds pre‐allocated based on population size within the geographical area, and then based on the number of staff per NHS trust. NHSCT are clearly aware of inequalities in need, but also in member charities' ability and experience in grant application. As one director put it: ‘so you've got some money going out equally and some money going out equitably’ (interview 2021006). In fact, some member charities voiced concerns about how funds collected locally are re‐distributed nationally and even described having ‘a bit of a running battle with NHS Charities Together for just who gets the money’ (focus group CS2.3).

These decisions about how to spend the proceeds of national fundraising campaigns are significant given the historic unevenness of NHS charity development across the UK. Distributing by staff head count alone is less equalising than an approach which, for example, takes population deprivation into account, but it enabled even NHS organisations where there was not a developed charity with professional charity staff to benefit. Moving forward, NHSCT is focusing on developing capacity among the membership, combined with a gradual shift towards more competitive schemes:

So we will have less money as we go forward, so probably more competitive grant making rather than just distribution of funds, a more strategic focus. Our next grant round … is about actually spending some money on the charities to develop themselves, to make them more robust. (interview 2021006)

Notably, responsibility for how the money was spent and projects implemented remained devolved to a local charity level. NHSCT ‘weren't overly prescriptive’ by their own accounts, instead emphasising that funded projects depended on perceived local needs. While this non‐prescriptive approach may have avoided tensions between national and local decision‐makers, it also raised anxieties over accountability and the need to closely evaluate local expenditure and impact. Another trustee stated that ‘we need to be absolutely convinced that if we've given these people six figure sums, it's being well spent’ (interview 2021004). The strategic move towards more competitive grants is likely to increase pressures on charities to demonstrate impact, but may also reinforce existing inequalities.

A second consequence of the successful COVID appeal was the extent to which the campaign gave NHSCT a seat at the table as an acknowledged national charity partner for the NHS. This was something actively requested by NHSCT leadership: ‘I'd already, as I said, got this very good relationship with NHS England by that point—so I asked “are we the National Appeal” and they went yes. So we set off from there.’ (NHSCT Chief Executive, quoted in Gorsky & Arnold‐Forster, 2024). In a crowded market of health charities, many of which are longstanding collaborators with the NHS, NHSCT use NHS branding carefully, but highly effectively to build a sense of them as ‘the’ charity partner of the NHS (Möller & Abnett, 2023). The NHSCT website is now presented more as a national charity which collaborates with local charities, than as a membership association: ‘we're the national independent charity caring for the NHS’ (NHS Charities Together, 2023). This excerpt from the website, and an associated graphic on the page which represents cash flows into and through NHSCT and its members, to the NHS, identifies some of the tensions that NHSCT navigates in its charitable mission as both membership association for local charities *and* national fundraiser in its own right.

### 4.4 National charitable fundraising in the NHS beyond COVID‐19

For official NHS England resources for the NHS's 75th birthday, donating money to an NHS charity was listed as one of the key ways for people to show their support for the service, alongside working for it, volunteering, giving blood or joining the organ donor register, or getting involved in research projects (NHS England, 2022). Since COVID, it is notable that national fundraising from NHSCT has been somewhat muted. Appeals have focused specifically on staff wellbeing: an issue with potential political sensitivities in a context of industrial action across multiple staff groups. The political climate has also meant that national fundraisers now have to ‘tread carefully’ as one senior trustee explained:

It's tricky at the moment. I think this year there's a lot of tension because of the strikes and because of the bitterness there is among some of the staff, so we're treading very carefully, not expecting too much, because you can't have people saying they're going out on strike on one hand and the next day saying, oh, come and have a jolly tea party, it's a bit tricky. (interview 2023004)

Even where the Big Tea has gone ahead, it has often been reinterpreted as part of wider staff support activities, rather than operating with an aggressive fundraising logic:

we're combining ours with a gratitude event for trust staff on each site and I think that's a good way to do it. It's the 75th birthday, let's say thank you without saying, give us lots of money, because I just don't think now is the time to be doing that. (interview 2023004)

By contrast, corporate partnerships with big national retailers and supermarkets are still progressing. Indeed, access to corporate brand partnerships is one of the main advantages for member charities without a national profile who are otherwise unlikely to receive support from major donors.

Yet, this balancing act between national and local fundraising is not without challenges. While demonstrating the extent to which NHSCT now has a ‘seat at the table’ in policy terms, expansion of fundraising at the national level is perceived to be in tension with ambitious, but newer NHS charities. A nationalised vision of popular support for the NHS can clash with a competitive ecosystem of local NHS charities. Furthermore, local members often argue that local loyalties, rather than NHS or NHSCT branding, make for more effective appeals. Fundraisers emphasised the importance of local causes and existing relationships with local businesses or community champions.

If I was there with NHS Big Tea, I can quite confidently say we wouldn't get anywhere near the money because it just doesn't say what it is, it doesn't say where the money's going. When you say to people, this is going to help build a new chemotherapy day unit in your local hospital, they're like going out and getting tenners out of the bank cash machine. (focus group CS2.3)

Such persistent localisms in fundraising may be at odds with national ambitions and vision for a unified campaign based on corporate partnerships. The considerable diversity among NHS charities, their established fundraising models and varying geographical needs may therefore compound existing inequalities in fundraising, and complicate the potential of future national campaigns.

## 5 CONCLUSION

Analysing the risks and potential of private, commercial finance in the NHS is a large and well‐established branch of scholarship (Exworthy, Lunt, et al., 2023; Hellowell & Pollock, 2009; Hodgson et al., 2022; Powell, 1996; Ruane, 1997). By contrast charitable funding of the NHS has, since a flurry of analyses in the wake of 1980s liberalisation (Lattimer, 1996; Pharoah & Mocroft, 2001), gone largely unexamined in social policy scholarship, or featured only as a footnote in analyses of broader processes of privatisation. This neglect is explained in part by the relative modesty of charitable fundraising for the NHS in crude financial terms: even the £150 million raised by NHS Charities Together's Urgent COVID‐19 appeal is dwarfed by the daily budget of the NHS in England alone. Nonetheless charitable funding in the UK NHS goes well beyond the notion of ‘nice extras’, with charitable finance contributing to clinical equipment (Abnett et al., 2023), buildings (Lattimer, 1996), and (by funding clinical research) creating new patient pathways (Viney et al., 2022).

However charitable funds also offer different actors power, as well as merely buying ‘things’. These funds provide discretionary money which can provide some freedom for organisations, especially during periods of scarcity and/or tight central control. The sums are subject to less national direction than other portions of the NHS budget and can therefore be disproportionately significant in creating either locally responsive, or problematically uneven, services (depending on one's viewpoint). Second, charitable money can act as a visible show of broader support from the public. While this might be used to galvanise individual hospitals within the NHS, it has also been mobilised politically at national scale. In early debates in the 1940s, desire to prevent fundraising by individual hospitals can be understood as a form of branding, claiming NHS ownership and financing of institutions by making a decisive break with their voluntary past. Different rules for regional and even national campaigns suggest that a more general objection to the principle of fundraising for healthcare was not at issue. More recently, NHS England's suggestion of a major national campaign as part of commemorations of the 70th anniversary of ‘the appointed day’, may have served as a useful reminder of public affection for the NHS in budget negotiations with Government.

By tracing debates about charitable fundraising back to the inception of the NHS, this paper demonstrates that the persistence of charitable money within the NHS since 1948 has been accompanied by shifts in conceptualisations of its role. This has oscillated between policy tolerance of local discretionary fundraising in a laissez faire fashion, and the development of a more nationally‐planned input to a traditional vision of the NHS as ‘a distinct, unified nationwide economy independent of business, designed to meet social needs rather than to maximise profit’ (Tudor Hart, 2006). In the early decades of the NHS, regional or national appeals were more acceptable for hospital staff and committee members than local fundraising, as formerly voluntary hospitals were absorbed into the new NHS. There followed from the 1980s the significant expansion of charitable fundraising by some NHS organisations, in a highly uneven fashion across the country. The 2018 advent of significant national fundraising in the NHS augured a shift in position from charities' somewhat ambivalent response to national fundraising for hospitals in the early years of the NHS, to an ‘official’ national campaign promulgating broader cultural intensification of the idea of the NHS as a singular, and uniquely British, entity (Stewart, 2023). A combination of NHS England's strategic desire to demonstrate public support for the NHS, with the increasingly professionalised expertise of charitable fundraisers, has had rippling effects across the sector. NHS Charities Together remains a membership organisation which also conducts its own fundraising, and it does so as the ‘official’ charity partner of the NHS. Both the business of NHS ‘birthday’ celebrations, and national fundraising more generally, have focused on expanding opportunities for ‘popular investment’ in the NHS (Bivins & Thomson, 2023), although they have additionally created some lucrative opportunities for corporate engagement.

Charity has thus played a role in the cultural intensification of a unitary and centralised vision of ‘the NHS’ in recent societal and political debates in the last decade (Stewart, 2023), despite the ongoing fragmentation of the service via devolution, and, in England, structural reorganisations and the internal market. The twists and turns of charitable fundraising in the NHS demonstrate Reich's argument that charity is ‘not an invention of the state, but ought to be viewed as an artefact of the state’ (Reich, 2020, p. 26). The role of charity within the mixed economy of the UK's healthcare system has changed over time in response to the changing NHS, even with few formal policy interventions in the form of legislation. With the exception of the 1980s liberalisation of local fundraising, changes in the role of charity in the NHS have not been coherently driven by Government as part of a broader policy agenda. Significant shifts identified above have been predominantly reactive; responding to initiatives from key actors within the NHS and from charity professionals. The growth of formal charity regulation has also influenced the sector to a greater extent than Government White Papers (Möller & Abnett, 2023). Despite near constant anxieties about sustainably financing the NHS, this has been an area of policy and practice deeply neglected by health policymakers and Governments for the last 40 years. At the time of writing it is not clear whether the unifying work of the COVID‐19 Urgent Appeal was a pandemic aberration, or the advent of a more collective approach to charitable funding for the NHS, perhaps encouraged by broader policy moves in England towards integrating across organisations (Sanderson et al., 2023). Future research should continue to expand our understanding of charitable fundraising in healthcare, including by exploring public perspectives on the appropriate role of such funding streams, and investigating the material difference made by charitable spending in the NHS across the UK.

## Data Availability

The data that support the findings of this study are available from the corresponding author upon reasonable request.

## Footnotes

^1^
National is a complex term as regards health policy in the UK. In this paper, we distinguish between local (i.e., at the level of hospital, Trust or Board) and national (meaning UK‐wide). Where points are specific to the distinctive legal and politics contexts of England, Scotland, Northern Ireland or Wales, we will state this.
